# Changes of starch and sucrose content and related gene expression during the growth and development of Lanzhou lily bulb

**DOI:** 10.1371/journal.pone.0262506

**Published:** 2022-01-11

**Authors:** Weitai Li, Dengjing Huang, Bo Wang, Xuemei Hou, Rui Zhang, Mei Yan, Weibiao Liao

**Affiliations:** College of Horticulture, Gansu Agricultural University, Lanzhou, China; Bangabandhu Sheikh Mujibur Rahman Agricultural University, BANGLADESH

## Abstract

As the main forms of carbohydrates, starch and sucrose play a vital role in the balance and coordination of various carbohydrates. Lanzhou lily is the most popular edible lily in China, mainly distributed in the central region of Gansu. To clarify the relationship between carbohydrate metabolism and bulb development of Lanzhou lily, so as to provide a basis for the promotion of the growth and development in Lanzhou lily and its important economic value, we studied lily bulbs in the squaring stage, flowering stage, half withering stage and withering stage. The plant height, fresh weight of mother and daughter bulbs continued to increase during the whole growth period and fresh weight of stem and leaf began to decrease in the half withering stage. The content of starch, sucrose and total soluble sugar in the lily mother bulb accumulated mostly in the flowering, withering and half withering stages, respectively. Starch, sucrose and total soluble sugar accumulated in the daughter bulb with the highest concentration during the withering stage. In the transcription level, sucrose synthase (*SuSy1*) and sucrose invertase (*INV2*) expressed the highest in squaring stage, and the expression was significantly higher in the mother bulb than in the daughter bulb. In flowering stage, the expression levels of soluble starch synthase (*SSS1*), starch-branching enzyme (*SBE*) and adenosine diphosphate-glucose pyrophosphorylase (*AGP1*) genes were higher in the mother bulb than in the daughter bulb. Altogether, our results indicate that starch and sucrose are important for the bulb growth and development of Lanzhou lily.

## Introduction

Lanzhou lily (*Lilium davidii* var. *unicolor*), a perennial herb, is a variety of *Lilium davidii*. Lanzhou lily is famous for its large, white and delicate taste bulbs, which is rich in minerals, trace elements, amino acids and other nutrients [1]. It is mainly grown in Gansu Province, China and is a very important agricultural plant with high economic value [2,3]. As a traditional medicinal plant and popular edible vegetable bulb, Lanzhou lily also has the function of clearing away heat and removing toxic, nourishing the lungs, anting cancer and enhancing human immunity [4,5].

The formation and robust development of bulb are key factors to maintain the normal life of Lanzhou lily. As the main part to store nutrients (mainly carbohydrates), bulbs have an important effect on the growth of Lanzhou lily [6]. Chlorophyll content in plant leaves is an important factor affecting dry matter synthesis. Meanwhile, carbohydrate is the main product of photosynthesis, and its accumulation is the basis of bulb enlargement [7,8]. The carbohydrates in the bulbs are regulated by both the aboveground and underground parts of lily plants. During the day, leaves fix the carbohydrates and consume them at night to support plant photosynthetic metabolism and growth [9]. More than a dozen of enzymes, such as sucrose synthase (SuSy) and invertase (INV), sucrose phosphate synthase (SPS), soluble starch synthase (SSS), starch-branching enzyme (SBE), adenosine diphosphate-glucose pyrophosphorylase (AGPase) and granule-bound starch synthase (GBSS), are involved in carbohydrate metabolism process [10].

Starch is an important form of carbohydrate storage in the bulb of Lanzhou lily. Starch consists of two glucose polymers: amylose and amylopectin [11]. The enzymes involved in starch synthesis mainly include SSS, SBE, AGPase and GBSS [12]. Previous study showed that the activities of these enzymes are usually positively associated with starch accumulation in “sink” organs [6]. Actually, the whole growth and development process of Lanzhou lily is actually the accumulation process of starch [13]. Studies have shown that the degradation of starch in lily bulbs is positively correlated with the increase of sucrose [14,15], which is the main sugar in the long-distance transport of sugar transporters from the reservoir tissues to the source tissues [16]. In general, sucrose not only serves as a carbon source to provide nutrients for plant growth and development, but also participates in the signal transduction process in plants as a signal substance [17]. SuSy and INV can regulate the participation of sucrose in starch synthesis [18]. They are present in a variety of cellular forms, making them important for the use of sucrose at different stages of plant growth and development [19]. As one of the key enzymes in regulating sucrose metabolism in plants, SuSy can mobilize sucrose to participate in structure composition and storage, tissue and cell metabolism, and regulate plant growth process, including providing substrate and energy for the synthesis of starch, cellulose and other substances [20,21]. In addition, the metabolism of starch and sucrose is closely related to soluble sugar, and their metabolic process is relatively complex.

Until now, the studies on Lanzhou lily mainly focused on abiotic stress response [2,3], plant regeneration [22], continuous cropping obstacles [23], multiple virus infections [24,25], the main constituents in bulbs [26], and so on. But there are few studies on the physiological and biochemical changes during Lanzhou lily growth and development, especially on the gene expressions related to carbohydrate metabolism during the development of lily bulbs. Thus, we studied the carbohydrate content in four different organs of Lanzhou lily after the seedling stage, and combined the gene expressions of carbohydrate in bulbs, in order to provide theoretical basis for clarifying the relationship between carbohydrate metabolism and bulb development in Lanzhou lily.

## Materials and methods

### Plant materials

The potted Lanzhou lilies (*Lilium davidii* var. *unicolor*) from Lanzhou city, Gansu Province, China were used as the materials in this study. The experiment was carried out in the greenhouse in Gansu Agricultural University, Lanzhou, China from April to December 2020. The healthy and single-headed bulbs weighing about 26 g were selected and planted in each pot with vermiculite and perlite (3:1). The potted lily bulbs were cultivated in the day/night condition of 25/18°C for 14/10 h under the natural sunlight. The conventional field management and no fertilization were carried out during the growth period. After planting, we studied the squaring stage (60 d), flowering stage (70 d), half withering stage (90 d) and withering stage (100 d) of lily growth. Finally, the mother bulbs, daughter bulbs, roots, stems and leaves were quickly frozen in liquid nitrogen and stored in a cryogenic refrigerator at -80°C.

### Morphological parameter determination

The length from the base of the stem to the growing point of the stem tip is the plant height, which was measured with a 1 m ruler. The fresh weight of Lanzhou lily plants was measured by destructive sampling. After cleaning the surface matrix, the Lanzhou lily plants were divided into mother bulb, daughter bulb, root, stem, and leaf. Then, the weight of them was weighed by electronic balance.

### Measurement of chlorophyll and carotenoid content and chlorophyll fluorescence parameters

According to the methods of Ghobadi et al. [27], fresh lily leaves (0.2 g) were used to determine chlorophyll content by a TU-1900 spectrophotometer (Shimadzu, Kyoto, Japan). The sample was soaked in 10 mL of 80% acetone in a dark place for 48 hours. After that, the chloroplast pigment extract was obtained, and the wavelength of TU-1900 spectrophotometer was adjusted to 663 and 645 nm for the determination of chlorophyll content. The chlorophyll and carotenoid content was calculated according to the following formula: chlorophyll a = 12.7OD_663_-2.59OD_645_, chlorophyll b = 22.9OD645−4.67OD_663_. Carotenoid = 1000*0.2*470–2.05Ca—114.8Cb/245.

The chlorophyll fluorescence parameters of Lanzhou lily plants were measured by the chlorophyll fluorescence imaging system (IMAG-PAM, Heinz Waltz, Germany) after being treated in the dark for about 30 minutes.

### Detection of soluble sugar content

Anthrone colorimetric method was used to determine the content of soluble sugar [28]. Fresh sample (0.2 g) was cut into pieces and boiled in 5 mL of distilled water. After 30 min, collecting the extract and repeated this process once again. The collected extract was adjusted to 25 mL and mixed well. Then, 0.125 mL extraction solution was suspended with 1.87 mL distilled water, 0.5 mL anthrone ethyl acetate reagent and 5 mL concentrated sulfuric acid. The mixture was kept in the boiling water for 1 min and then cooled to the room temperature. The soluble sugar content was detected by a TU-1900 spectrophotometer at 630 nm.

### Starch content analysis

The starch content was determined by the iodine colorimetric method refer to Kuai et al. [29]. Fresh sample (0.5 g) was firstly grounded with 2 mL distilled water and then 3.2 mL 60% perchloric acid. Above solution was collected and centrifuged at 5000 *g* for 5 min. The supernatant about 0.5 mL was mixed with 3 mL of distilled water and 2 mL of iodine reagent. The absorbance of the supernatant was measured at 660 nm.

### Sucrose content determination

The sucrose content was determined by anthrone spectrophotometry. About 1 g sample was grounded and extracted in 80% ethanol. The collection was firstly incubated at 80°C for 45 min, and then 0.4 mL of the extract was added into 200 μL of sodium hydroxide (2 mol⋅ L^-1^). The mixture was incubated in the boiling water for 5 min and then reacted with 2.8 mL of 30% hydrochloric acid and 1% resorcinol solution at 80°C for 10 min. The above cooled solution was used to measure the OD value at 630 nm.

### Quantitative real-time PCR

Total RNA was extracted by TRIzol method and with some modifications. The sample was ground into powder by adding liquid nitrogen and put into a centrifugal tube. TRIzol (1 mL) was added to lyse the cells, and then mixed with 200 μL chloroform and incubated for 5 min. The solution was centrifuged at 4°C, 12000 *g* for 15 min. Then, an equal volume of isopropanol was added and precipitated at -20°C for 1 hour. The supernatant was centrifuged at 12000 *g* at 4°C for 15 min and then washed by 1 mL 75% ethanol for twice (12000 *g*, 4°C for 30 s). The RNA was collected with 30–50 μL of RNase-Free ddH_2_O. The cDNA was synthesized by Evo M-ML V RT Premix for qPCR (Accurate Biotechnology, Hunan, China) according to the manufacturer’s instructions. The SYBR Green Premix *Pro Taq* HS qPCR Kit (Accurate Biotechnology, Hunan, China) was used for quantitative real-time PCR. The reaction conditions were as follows: 95°C for 30 s, 40 cycles of 95°C for 5 s, and 60°C for 34 s. *LoTIP1* was used as internal reference. All primer sequences were referred to Li et al. [10]. All experimental treatments have three replicates.

### Data analysis

SPSS statistical software (IBM Corp., Armonk, NY, USA) was used for statistical analysis. All the data were analyzed for differences among treatments using one-way ANOVA, Duncan’s was used to detect significant differences between treatments (*P* < 0.05).

## Results

### Plant height and fresh weight

**Fig 1:**
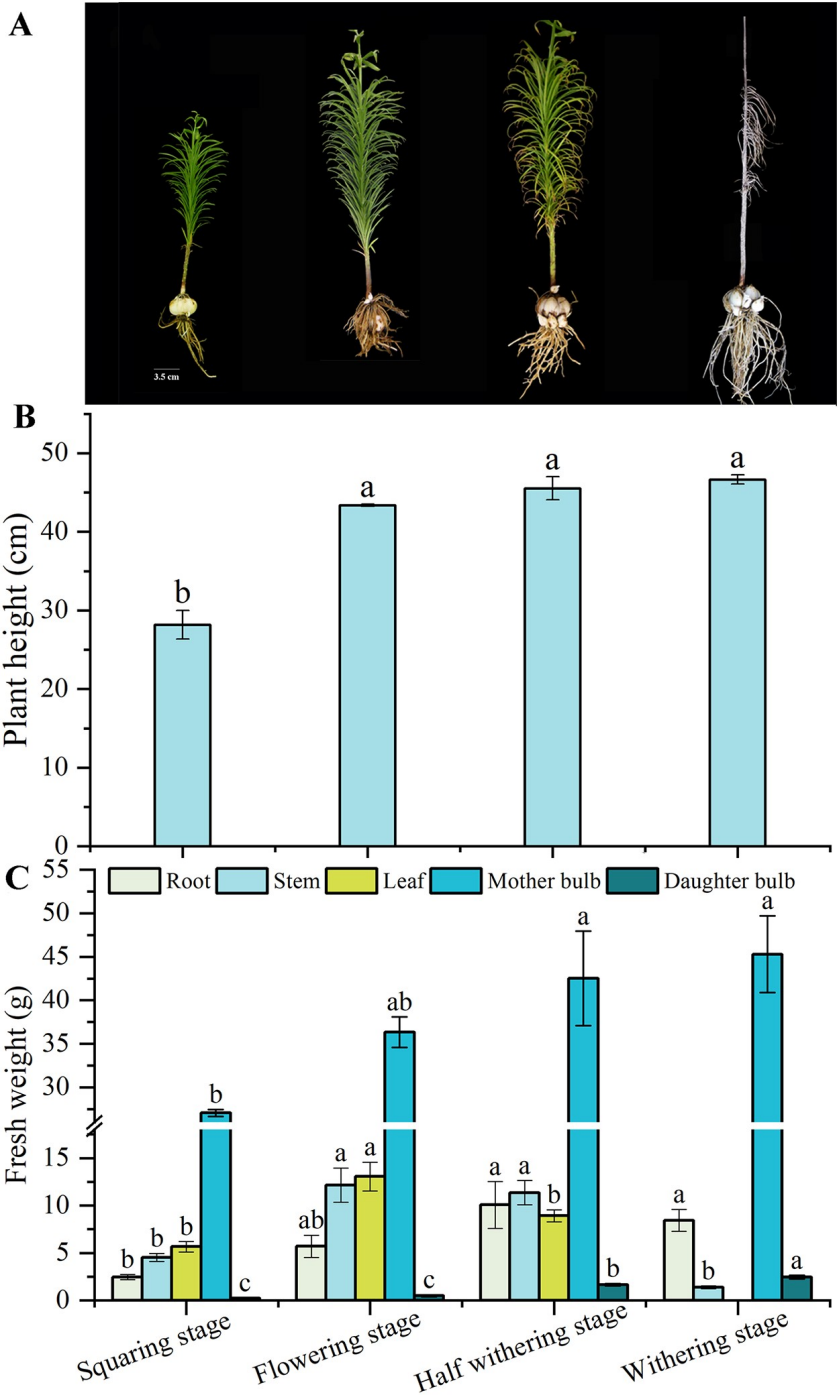
The morphologies (A), plant height (B) and fresh weight (C) during the growth of Lanzhou lily. Mean ± SE (n = 3), different letters indicate significant differences in different stages of the same organization.

In the budding stage, the scales of the mother bulbs were tight, and there was almost no small bulb produced. From the flowering stage, the daughter bulbs were gradually produced and obviously gradually enlarged in the next two stages, while the size of the mother bulbs does not change significantly (Fig 1A). And the branches and leaves grow from the middle of mother bulbs, making the scales of mother bulbs slightly loose. As shown in Fig 1B, the plant height was increasing gradually with time, and the increase was most significantly during squaring stage to flowering stage, but not significant after flowering. Similarly, the fresh weight of both daughter and mother bulbs were also increased gradually. The daughter bulb fresh weight increased significantly after flowering stage, which was differ to that of the mother bulb whose fresh weight increased gradually after flowering stage (Fig 1C). The fresh weight of root, stem and leaf all showed he trend of increase firstly and decreased afterward. The fresh weight of stem and leaf increased before flowering stage and then decreased. Root fresh weight begun decreasing after half withering stage.

### Chlorophyll and carotenoid content and chlorophyll fluorescence parameters

**Fig 2:**
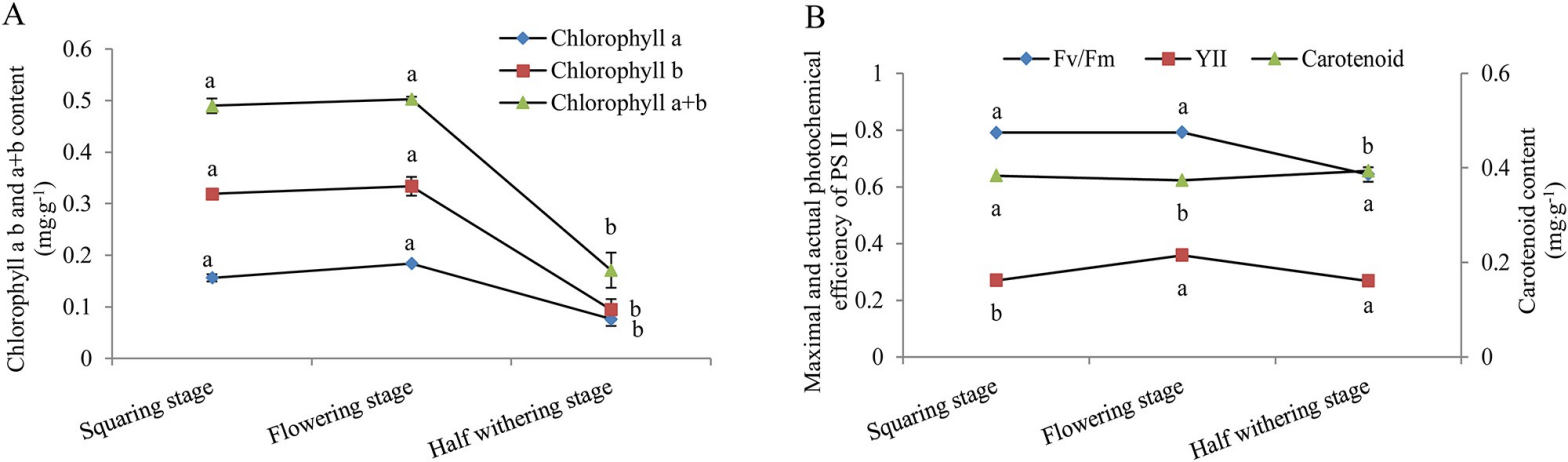
Changes of chlorophyll content (A), carotenoid content and chlorophyll fluorescence parameters (B) in leaves during the growth stage of Lanzhou lily. Mean ± SE (n = 3), different letters indicate significant differences in different periods of the same organization.

The content of chlorophyll a, b and a + b were slightly increased from the squaring stage to the flowering stage and without significant difference, but that in flowering stage and half withering stage decreased sharply from 0.18 mg⋅ g^-1^ to 0.07 mg⋅ g^-1^, 0.33 mg⋅ g^-1^ to 0.09 mg⋅ g^-1^ and 0.5 mg⋅ g^-1^ to 0.17 mg⋅ g^-1^, respectively (Fig 2A). The change trend of chlorophyll fluorescence parameters was consistent with chlorophyll content. The maximum photochemical efficiency (Fv/Fm) and actual photochemical efficiency (YII) increased slowly before flowering, and then began to decrease slowly (Fig 2B). However, the carotenoid content showed the opposite trends to chlorophyll content, and reached the lowest value (0.37 mg⋅ L^-1^) during the flowering stage.

### Carbohydrate content

**Fig 3:**
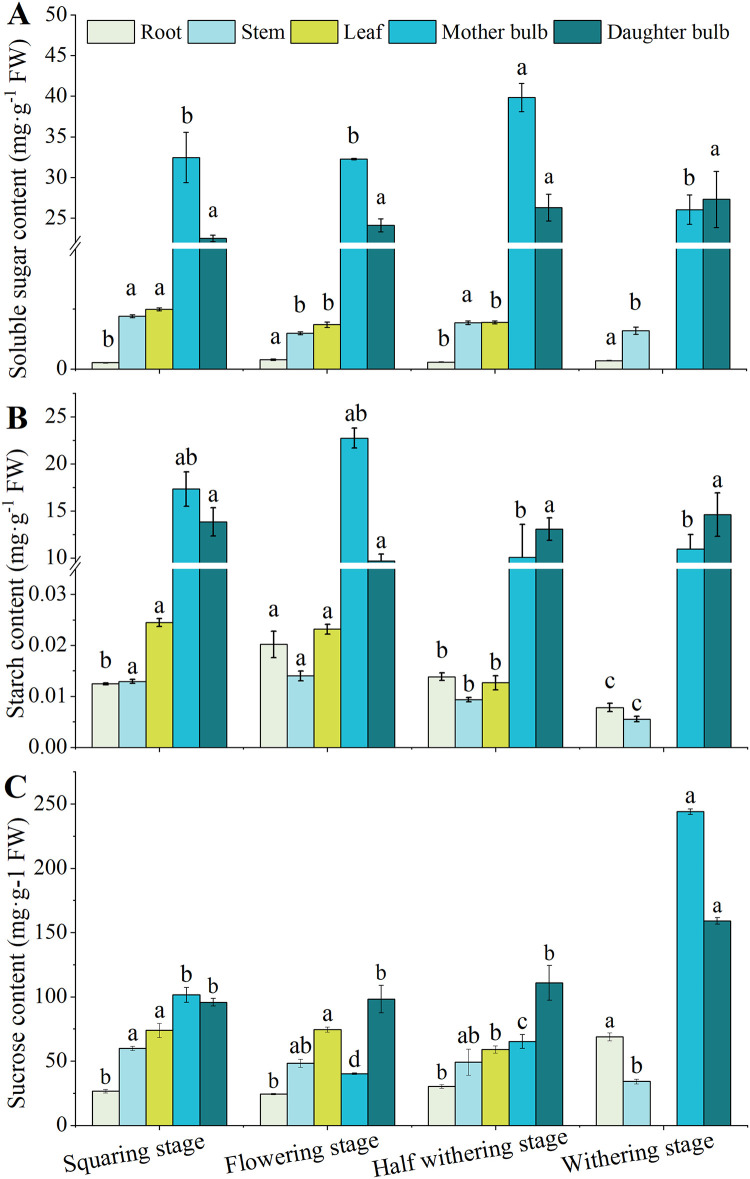
The carbohydrate content during the growth of Lanzhou lily. The soluble sugar content (A), starch sugar content (B) and sucrose content (C) in root stem leaf mother bulb and daughter bulb. Mean ± SE (n = 3), different letters indicate significant differences in different periods of the same organization.

The soluble sugar content of the daughter bulb was increased with the growth (Fig 3A). Differently, the soluble sugar content of the mother bulb, stem and leaf was lower in the flowering than those in the squaring stage. And then, the soluble sugar contents in these organs were firstly increased in the half-withered stage but decreased significantly in the withering stage. However, the soluble sugar content in root was opposite to that in mother bulb, stem and leaf. The starch content of mother bulb showed a trend of increased firstly, then decreased, and then increased again from the flowering stages, half withered stage and withering stage, respectively (Fig 3B). Differently, the starch content of daughter bulb decreased in flowering, and increased in the half-withered to withering stages. In root, the starch content was increased during the flowering stage, and then decreased gradually. However, the starch content of root, stem and leaf increased in the flowering stage, and then decreased in the latter two stages. The sucrose content of mother bulbs in the flowering stage was significantly lower than the squaring stage, and then continue increased to withering until maximum (Fig 3C). Meanwhile, the sucrose content of daughter bulbs was increasing gradually during the whole development. The sucrose content of stem decreased from the squaring to the flowering stage, increased to the half-withered stage, and then decreased to withering stage. The change trend of sucrose content in leaves was opposite to that in roots.

### Gene expression patterns of glucose metabolism-related enzymes in mother bulbs and daughter bulbs

**Fig 4:**
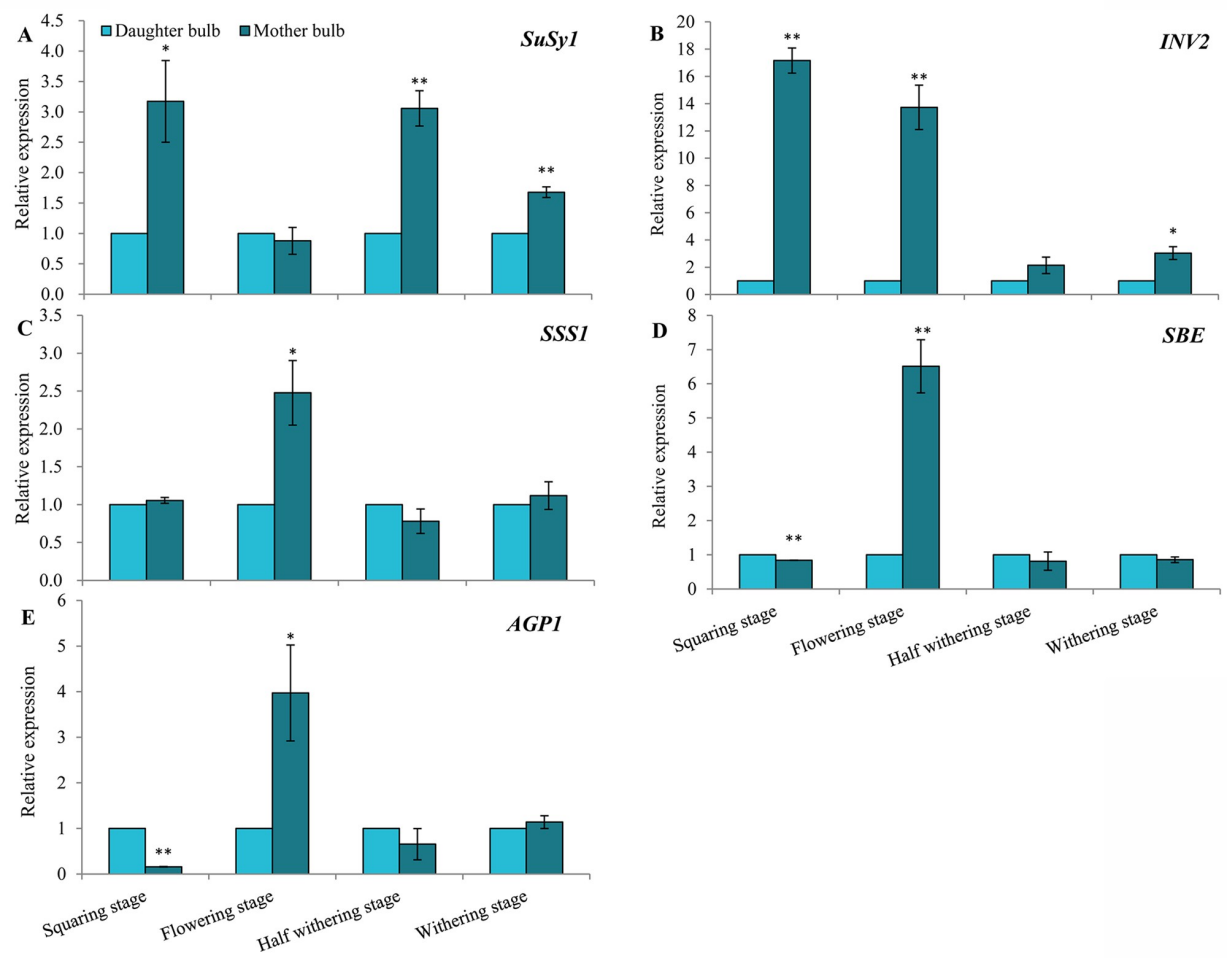
Expression profiles of five genes in mother bulbs and daughter bulbs of *Lilium davidii* var. *unicolor* by the quantitative real-time PCR. **A**: *SuSy1* (sucrose synthase 1); **B**: *INV2* (sucrose invertase 2); **C**: *SSS1* (soluble starch synthase 1); **D**: *SBE* (starch-branching enzyme); **E**: A*GP1* (Adenosine diphosphate-glucose pyrophosphorylase 1). Mean ± SE (n = 3). Asterisk indicate significant differences in different periods of the same organization.

In order to verify the gene expression patterns of starch and sucrose metabolism-related enzyme in mother and daughter bulbs, quantitative real-time PCR was used. The expressions of *SuSy1*, *INV2*, *SSS1*, *SBE* and *AGP1*. *SuSy1* were significantly higher in the mother bulbs in the squaring, half withering and withering stage than that in the daughter bulbs (Fig 4A). However, the expression level of *INV2* gene in mother bulbs was higher than that in daughter bulbs at all four stages. Among these stages, *INV2* expression levels in mother bulbs at squaring and flowering stages was 16.16 and 12.72 times higher than that in the daughter bulbs (Fig 4B). *SSS1*, *SBE* and *AGP1* gene expressions were higher in the mother bulbs at flowering stage than in the daughter bulbs, while stable in both the mother and daughter bulbs of squaring, half withering and withering stages, respectively (Fig 4C–4E). The expression of *SBE* and *AGP1* in mother bulbs at squaring stage were significantly lower than that in daughter bulbs. Conversely, *SBE* and *AGP1* were higher expressed in mother bulbs after squaring, and reached 1.31 and 1.28 times higher than daughter bulbs at flowering stage, respectively (Fig 4D and 4E).

## Discussion

As a famous food crop with more than 400 years history, Lanzhou lily is well-known throughout China [30]. During the growth and development, vegetative growth of the aboveground part of the plant is closely related to the quality and yield of Lanzhou lily bulbs. Since the buds firstly appeared, the plant height and the fresh weight of mother bulb and daughter bulb increased gradually, and increased most obviously from the squaring stage to the flowering stage (Fig 1). This is consistent with the study of Zou et al. [31], who found that the bulb weight of *Lilium longiflorum* could increase sharply during the early flowering period. The plant began to wither gradually during the half-withered stage, and the fresh weight of stems and leaves also began to decline. Chlorophyll, as the absorption, transfer and transformation of light energy in photosynthesis, plays a key role in plant photosynthesis and is one of the important indexes reflecting plant photosynthesis [32]. The chlorophyll content began to decrease sharply after flowering (Fig 2). This indicates that the synthesis of chlorophyll in lily mainly occurred in the flowering stage and may not occur in the bulb expansion stage.

Carbohydrates in lily bulbs are the main nutrients and energy sources for substance synthesis. Starch is an important form of carbohydrate storage in the bulbs of Lanzhou lily [33], and its metabolic characteristics are important for the formation and development of daughter bulb. Sucrose is the dominant form of soluble carbohydrates, which is responsible for the allocation of key carbon resources and the initiation of sugar signaling. Sucrose plays an important role in the morphological formation of daughter bulbs [34]. In our experiment, after squaring stage, the leaf function of Lanzhou lily plants was gradually improved, and the starch content in the bulb of Lanzhou lily was increased (Fig 3). At this phase, the development of the plant still consumed carbohydrates in the mother bulb, indicating that the bulb of Lanzhou lily at this stage was the plant ‘‘Metabolic pool”. After flowering, the starch content of the bulb decreases and the soluble sugar content increased (Fig 3), indicating that the starch is degraded into soluble sugar and provides carbon source and energy for bulb development. In half withering stage, plant withered gradually and its photosynthesis weakened, thus the starch content of the bulb also begins to be consumed and converted to sucrose. The content of sucrose was correspondingly increased significantly (Fig 3), suggesting that starch is the source of carbon skeleton for the synthesis of sucrose [20]. With the extension of the growth time, the soluble sugar and sucrose content in the daughter bulbs all increased gradually. Starting from the half withering stage, the starch content in the daughter bulbs was significantly higher than that in the mother bulbs. On the one hand, this may be due to plant consumes starch during the flowering and half withering stage, and on the other hand, it might transfer nutrients to the daughter bulbs and promote its growth.

So far, little research has been done on Lanzhou lily genes, and the genome database has not been published. The five starch and sucrose-related genes were studied in this paper. Both SuSy and INV are the main enzymes involved in sucrose decomposition. The difference is that the former can reversibly catalyze the metabolism of sucrose, while the latter can decompose sucrose in a one-way way [10,35]. From the analysis of gene expression, *SuSy1* and *INV2* decreased in the mother bulb during the flowering stage (Fig 4), this is consistent with the decrease of sucrose content (Fig 3). This result was the same to the work of Ahmeda et al. in cotton [36]. The expression *of INV2* was decreased significantly after flowering, suggesting that the flowering stage was the transition stage of bulbous expansion of Lanzhou lily [2]. Sucrose in mother bulbs is hydrolyzed to provide energy for starch synthesis and the formation and development of daughter bulbs [37]. The main form of starch in Lanzhou lily is amylopectin, which accounts for a large part of the total starch [38]. Starch metabolism is a complicated process, including starch biosynthesis, decomposition and transportation. A series of enzymes (SSS, SBE, AGPase, GBSS, etc.) that synthesize starch are synergistic [39]. In our study, genes about starch biosynthesis-related enzyme genes (*SSS1*, *SBE* and *AGP1*) expressed highest at the flowering stage (Fig 4), which was consistent with the starch content of the mother bulbs (Fig 3). Except the flowering stage, the expression level of starch synthesis-related enzyme gene in daughter bulb was higher than that in the mother bulb, and combining with the continuous increase in the fresh weight of small bulbs, we concluded that starch might play important roles in the growth and development of daughter bulb. And this was also reported by Shin et al. in *Lilium Oriental hybrid* ‘*Casablanca*’ and *Lilium Asiatic hybrid* ‘*Mona*’ [40].

## Conclusion

The results of this study suggested that after the squaring stage of Lanzhou lily, as the plant grows, both the mother and daughter bulbs gradually grow up. Since the growth and development of bulbs is a dynamic process, different carbohydrates have different expressions in different parts at different growth stages. The content of soluble sugars, starch and sucrose in mother/child bulbs was relatively highest compared to other organs. These carbohydrates, especially the interconversion between starch and sugar, provide important energy substances for the growth and development of bulbs. Thus, carbohydrates, especially starch and sugar play an extremely important role in the growth and development of Lanzhou lily bulbs in different periods.

## Supporting information

S1 Data (XLSX)

## Abbreviations

AGPase: Adenosine diphosphate-glucose pyrophosphorylase
GBSS: Granule-bound starch synthase
INV: Sucrose invertase
SBE: Starch-branching enzyme
SPS: Sucrose phosphate synthase
SSS: Soluble starch synthase
SuSy: Sucrose synthase

## References

[1] Zhang YB, Wang YJ, Meng J, Xie ZK, Wang RY. Development of an immunochromatographic strip test for rapid detection of lily symptomless virus. J Virol Methods. 2015; 220(000): 13–17. doi: 10.1016/j.jviromet.2015.03.021 25845624

[2] Li WM, Wang YJ, Zhang YB, Wang RY, Guo ZH, Xie ZK. Impacts of drought stress on the morphology, physiology, and sugar content of Lanzhou lily (*Lilium davidii* var. *unicolor*). Acta Physiol Plant. 2020; 42(8): 127. doi: 10.1007/s11738-020-03115-y

[3] Tian XH, Xie JM, Yu JH. Physiological and transcriptomic responses of Lanzhou Lily (*Lilium davidii*, var. *unicolor*) to cold stress. Plos One. 2020; 15(1): e0227921. doi: 10.1371/journal.pone.0227921 31971962 PMC6977731

[4] Huang DJ, Li WT, Mohammed MD, Huo JQ, Li CX, Wang CL, et al. Hydrogen Sulfide Reduced Colour Change in Lanzhou Lily-Bulb Scales. Postharvest Biol Tec. 2021; 176(000). doi: 10.1016/j.postharvbio.2021.111520

[5] Li WM, Wang YJ, Wei HL, Zhang YB, Guo ZH, Qiu Y, et al. Structural characterization of Lanzhou lily (*Lilium davidii* var. *unicolor*) polysaccharides and determination of their associated antioxidant activity. J Sci Food Agr. 2020; 100(15):5603–5616. doi: 10.1002/jsfa.10613 32608519

[6] Wu Y, Xia YP, Zhang JP, Du F, Zhang L, Zhou H. Low humic acids promote in vitro lily bulblet enlargement by enhancing roots growth and carbohydrate metabolism. Zhejiang Univ-Sci B. 2016; 17(11): 892–904. doi: 10.1631/jzus.B1600231 27819136 PMC5120231

[7] Wu Y, Sun MY, Zhang JP, Zhang L, Ren ZM, Min RH, et al. Differential Effects of Paclobutrazol on the Bulblet Growth of Oriental Lily Cultured In Vitro: Growth Behavior, Carbohydrate Metabolism, and Antioxidant Capacity. Plant Growth Regul. 2019; 38(2): 359–372. doi: 10.1007/s00344-018-9844-5

[8] Guidi L, Piccolo EL, Landi M. Chlorophyll fluorescence, photoinhibition and abiotic stress: does it make any difference the fact to be a C3 or C4 species? Front. Plant Sci. 2019; 10(000): 174. doi: 10.3389/fpls.2019.00174 30838014 PMC6382737

[9] Angeles-Núñez JG, Tiessen A. Arabidopsis sucrose synthase 2 and 3 modulate metabolic homeostasis and direct carbon towards starch synthesis in developing seeds. Planta. 2010; 232(3): 701–718. doi: 10.1007/s00425-010-1207-9 20559653

[10] Li XY, Wang CX, Cheng JY, Zhang J, Jaime AT, Liu XY, et al. Transcriptome analysis of carbohydrate metabolism during bulblet formation and development in *Lilium davidii* var. *unicolor*. BMC Plant Biol. 2014; 14: 358. doi: 10.1186/s12870-014-0358-4 25524032 PMC4302423

[11] Gao MP, Zhang SW, Luo C, He XH, We SL, Jiang W, et al. Transcriptome analysis of starch and sucrose metabolism across bulb development in *Sagittaria sagittifolia*. Gene. 2018; 649(000): 99–112. doi: 10.1016/j.gene.2018.01.075 29374598

[12] Tetlow IJ, Emes MJ. Starch Biosynthesis in the Developing Endosperms of Grasses and Cereals. Agronomy. 2017; 7(4):81. doi: 10.3390/agronomy7040081

[13] Mukerjea R, Yu L, Robyt JF. Starch biosynthesis: mechanism for the elongation of starch chains. Carbohyd Res. 2002; 337(11): 1015–1022. doi: 10.1016/s0008-6215(02)00067-8 12039542

[14] Mukherjee S, Liu A, Deol KK, Kulichikhin K, Stasolla C, Brûlé-Babel A, et al. Transcriptional coordination and abscisic acid mediated regulation of sucrose transport and sucrose-to-starch metabolism related genes during grain filling in wheat (*Triticum aestivum* L.). Plant Sci. 2015; 240(000): 143–160. doi: 10.1016/j.plantsci.2015.09.010 26475195

[15] Du F, Fan JM, Wang T, Wu Y, Grierson D, Gao Z, et al. Identification of differentially expressed genes in flower, leaf and bulb scale of *Lilium oriental hybrid* ‘*Sorbonne’* and putative control network for scent genes. Bmc Genom. 2017; 18: 899. doi: 10.1186/s12864-017-4303-4 29166855 PMC5700745

[16] Ruan YL. Sucrose metabolism: gateway to diverse carbon use and sugar signaling. Annu. Rev. Plant Biol. 2014; 65(000): 33–67. doi: 10.1146/annurev-arplant-050213-040251 24579990

[17] Gao SQ, Zhu Y, Zhou LY, Fu XF, Lei T, Chen QB, et al. Sucrose signaling function on the formation and swelling of bulblets of *Lilium* sargentiae E.H. Wilson. Plant Cell Tiss Org. 2018; 135(1): 143–153. doi: 10.1007/s11240-018-1451-4

[18] Durand M, Mainson D, Porcheron B, Maurousset L, Lemoine R, Pourtau N. Carbon source-sink relationship in *Arabidopsis thaliana*: The role of sucrose transporters. Planta. 2018; 247(3): 587–611. doi: 10.1007/s00425-017-2807-4 29138971 PMC5809531

[19] Gu JH, Zeng Z, Wang YR, Lyu YM. Transcriptome Analysis of Carbohydrate Metabolism Genes and Molecular Regulation of Sucrose Transport Gene *LoSUT* on the Flowering Process of Developing Oriental Hybrid Lily ‘*Sorbonne’* Bulb. Int J Mol Sci. 2020; 21(9): 3092. doi: 10.3390/ijms21093092 32349427 PMC7247698

[20] Jiang L, Yu X, Qi X, Yu Q, Deng S, Bai B, et al. Multigene engineering of starch biosynthesis in maize endosperm increases the total starch content and the proportion of amylose. Transgenic Res. 2013; 22(2):1133–1142. doi: 10.1007/s11248-013-9717-4 23740205

[21] Shin KS, Chakrabarty D, Paek KY. Sprouting rate, change of carbohydrate contents and related enzymes during cold treatment of lily bulblets regenerated in vitro. Sci Hortic. 2002; 96(1–4): 195–204. doi: 10.1016/S0304-4238(02)00087-0

[22] Xu LF, Ma FW, Liang D. Plant regeneration from in vitro cultured leaves of Lanzhou lily (*Lilium davidii* var. *unicolor*). Sci Hortic. 2009; 119(4): 458–461.

[23] Hua CP, Xie ZK, Wu ZJ, Zhang YB, Guo ZH, Qiu Y, et al. The Physiological and Biochemical Effects of Phthalic Acids and the Changes of Rhizosphere Fungi Diversity under Continuous Cropping of Lanzhou Lily (*Lilium davidii* var. *unicolor*). Hortic Sci. 2019; 54(2): 253–261. doi: 10.21273/HORTSCI13527-18

[24] Zhang YB, Wang YJ, Xie ZK, Yang G, Guo ZH, Wang L. The occurrence and distribution of viruses infecting Lanzhou lily in northwest. Crop Prot. 2018; 1109(000): 73–76. doi: 10.1016/j.cropro.2018.02.028

[25] Yan ZQ, He XF, Guo K, Li XZ, Yang XY, Jin H, et al. Allelochemicals from the rhizosphere of Lanzhou lily: Discovery of the autotoxic compounds of a bulb crop. Sci Hortic. 2019; 250(000): 121–126. doi: 10.1016/j.scienta.2019.02.038

[26] Wang FX, Wang W, Niu XB, Huang YL, Zhang J. Isolation and Structural Characterization of a Second Polysaccharide from Bulbs of Lanzhou Lily. Appl Biochem Biotech. 2018; 186(3):535–546 doi: 10.1007/s12010-018-2750-2 29663128

[27] Ghobadi M, Taherabadi S, Ghobadi ME, Mohammadi GR, Saeid JH. Antioxidant capacity, photosynthetic characteristics and water relations of sunflower (*Helianthus annuus* L.) cultivars in response to drought stress. Ind Crop Prod. 2013; 50(000):29–38. doi: 10.1016/j.indcrop.2013.07.009

[28] Du YL, Zhao Q, Chen LR, Yao XD, Zhang W, Zhang B, et al. Effect of drought stress on sugar metabolism in leaves and roots of soybean seedlings. Plant Physiol Bioch. 2020; 146(000):1–12. doi: 10.1016/j.plaphy.2019.11.003 31710920

[29] Kuai J, Liu ZW, Wang YH, Meng YL, Chen BL, Zhao WQ, et al. Waterlogging during flowering and boll forming stages affects sucrose metabolism in the leaves subtending the cotton boll and its relationship with boll weight. Plant Sci. 2014; 223(000):79–98. doi: 10.1016/j.plantsci.2014.03.010 24767118

[30] Cao X, Du YL, Zhang XS, Li HY, Guo SJ, Hou D, et al. First Report of Leaf Blight Disease on Lanzhou Lily (*Lilium davidii* var. *unicolor*) Caused by Botrytis cinerea in China. Plant diseae. 2019; 02: 0374. doi: 10.1094/PDIS-02-19-0374-PDN

[31] Zou HG, Ning YF, Jiang RL. Dynamic changes of development of characters in *Lilium longiflorum*. Northern Hort. 2003; 60–61. doi: 10.21273/HORTSCI11463-16

[32] Zhang YJ, Yan F, Gao H, Xu YZ, Guo YY, Wang E, et al. Chlorophyll Content, Leaf Gas Exchange and Growth of Oriental Lily as Affected by Shading. Plant Physiol. 2015; 62(3): 334–339. doi: 10.1134/S1021443715030206

[33] Chang L, Xiao YM, She LF, Xia YP. Analysis of gene expression and enzyme activities related to starch metabolism in *Lycoris sprengeri* bulbs of different sizes. Sci Hortic-Amsterdam. 2013; 161(000): 118–124. doi: 10.1016/j.scienta.2013.07.005

[34] Yang PP, Xu LF, Xu H, Tang YC, He GR, Cao YW, et al. Histological and Transcriptomic Analysis during Bulbil Formation in *Lilium lancifolium*. Front. Plant Sci. 2017; 8: 01508. doi: 10.3389/fpls.2017.01508 28912794 PMC5582597

[35] Li GH, Pan JF, Cui KH, Yuan MS, Hu QQ, Wang WC, et al. Limitation of unloading in the developing grains is a possible cause responsible for low stem non-structural carbohydrate translocation and poor grain yield formation in rice through verification of recombinant inbred lines. Front. Plant Sci. 2017; 8: 01369. doi: 10.3389/fpls.2017.01369 28848573 PMC5550689

[36] Ahmeda M, Akhtarb S, Fanglua M, Hasanb MM, Shahidb AA, Yanangb X, et al. Sucrose Synthase (SuSy) Gene Expression: An Indicator for Cotton Fiber Initiation and Early Development. Russ J Plant Physl. 2019; 66(1): 41–49. doi: 10.1134/S1021443719010023

[37] Zhang CS, Zhang HW, Zhan ZX, Liu BJ, Chen ZT, Liang Y. Transcriptome Analysis of Sucrose Metabolism during Bulb Swelling and Development in Onion (*Allium cepa* L.). Plant Sci. 2016; 7: 01425. doi: 10.3389/fpls.2016.01425 27713754 PMC5031786

[38] Wang HR, Yao JY, Zhang TT, Cui N, He S, Zhang R, et al. Relationship between neutral invertase activity and sugar contents in tomato fruit and its functional prediction analysis. Biotechnol. J. Int. 2017; 20(1): 1–6.

[39] Shen JL, Wang Y, Shu S, Jahan MS, Zhong M, Wu JQ, et al. Exogenous putrescine regulates leaf starch overaccumulation in cucumber under salt stress. Sci Hortic-Amsterdam. 2019; 253(000): 99–110. doi: 10.1016/j.scienta.2019.04.010

[40] Shin KS, Chakrabarty D, Paek KY. Sprouting rate, change of carbohydrate contents and related enzymes during cold treatment of lily bulblets regenerated *in vitro*. Sci Hortic. 2002; 96(1). doi: 10.1016/S0304-4238(02)00087-0

